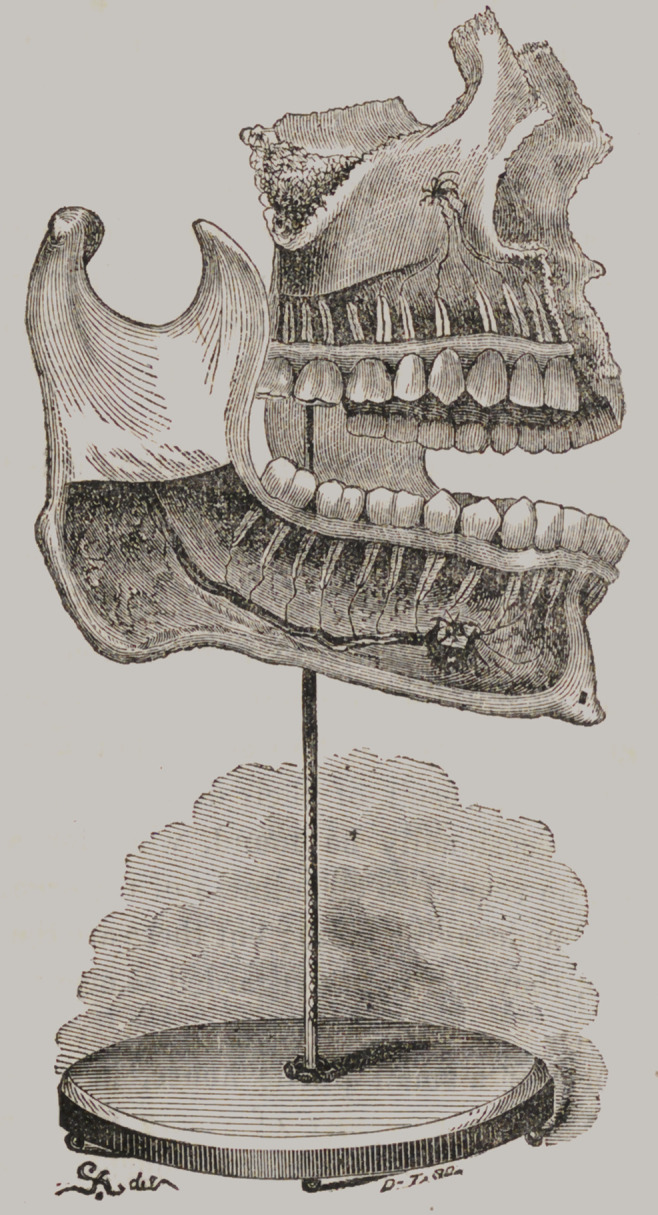# Physiological Observations on the Teeth

**Published:** 1859-02

**Authors:** T. E. St. John


### THE DENTAL REPORTER.

VOL. 1. FEBRUARY, 1859. No. 4.

### PHYSIOLOGICAL OBSERVATIONS ON THE TEETH.

BY PROF. T. E. ST. JOHN.

THE CAUSES OF DECAY.—One of the greatest evils that "flesh is heir to," is the tendency to decay of the dental organs. Why is it? What induces this premature degeneration in structures that for hardness and density are not surpassed by any in the body? It is a lamentable fact that scarcely one in ten of the human family arrive at the age of maturity, without suffering more or less the tortures of decayed and decaying teeth. It can not, certainly, all be attributed to the faults and excesses of the parent. It can not be supposed that the present generation are so exempt from misdemeanors or transgressions, as to be compelled to suffer the penalties of *hereditary* diseases alone. No, there is a cause; in fact it might be said there are many causes for this most distressing ill of the human subject, that can not be referred to any circumstances existing previous to the birth of the sufferer. I have before noticed the best means calculated to induce and maintain a healthy denture, and it is the object of this paper, to consider the *causes of decay* in the permanent teeth.

These are various, if we are to believe the assertions of different writers upon the subject. Thus, acids, alkalies, climatic influences, the mode of living, the use of sugar, uncleanliness, etc., etc., are presented as a few of the prolific causes of this malady. In noticing the effect of these upon the teeth, I shall endeavor to ascertain how much they are entitled to the censure they have received at the hands of the writers who have spoken of their demerits.

ACIDS.—Dr. Rossi says, that the change in teeth known as caries, is a *chemical* phenomenon, and is due to a single cause; the action of *one, or several acids*. In examining the composition of the tooth, it is found to contain considerable quantities of alkaline earths, which are capable of combining with various acids, when they are placed under favorable circumstances. All acids are not capable of acting upon the teeth, neither is the effect of those that are injurious, similar in all cases. Acids may be present in the mouth from a number of sources; they may be secreted by the subject, in the saliva and the buccal mucus, especially if the individual be affected with any disease of the salivary glands, or even when suffering from any constitutional malady, the secretions may be so changed in quality, or character, as to become corrosive, or destructive to the teeth, from the excess of acid they may contain. Acids may be introduced into the mouth with the substances taken as food, or may be taken alone, either as drinks or medicines. Particles of the food will remain in contact with the teeth, because of the absence of ordinary efforts of cleanliness, a sufficient length of time for decomposition or putrefaction to take place, which will generate acids in considerable quantities, some of which may be peculiarly active, producing their deleterious effects very rapidly.

It is not to be supposed that a solution of any ordinary acid of the strength in which it is found in the mouth, will materially effect the sound tooth, but if by mechanical violence or other means, the outer coating of enamel becomes removed, it will be observed to act with considerable energy. It is a common remark of all who have any experience in the matter, that when the cavity is once formed, the tooth soon beçomes worthless, from the rapid increase of the carious portion.

An examination of the positions of the decayed part would indicate that the decomposition of food and consequent formation of acid was one of the most common causes of caries. It is found on the approximal surface where the material may easily accumulate; it is also found upon the top of the crown in the depressions where the enamel is developed in small quantity.

There are certain conditions of the dental structure, that favor the destructive action of acids, which come in contact with them, viz: the quality of the dental structure, which from the presence of disease in the subject, or from an hereditary taint which may exist, owing to circumstances mentioned in the previous article in the REPORTER, upon the means of *securing* good teeth. The form of the teeth may favor the accumulation of foreign substances, by having a number of large depressions, or being placed in the dental arch in an awkward and irregular manner. Mechanical injuries, such as will result from the use of metalic tooth-picks, the abuse of the teeth by cracking nuts, biting off nails and the like, favor the action of the acids by injuring the enamel and thus allowing them to come in contact with the less protected portions of the tissues.

Thus it will be seen that there are many circumstances existing, which would favor the action of acids upon these organs. Is the presence of this substance injurious? If a tooth be macerated for a short time, in a weak solution of nitric, sulphuric or muriatic acid, it will be found to present the appearances of decay, the tissues will become colored or broken down and disintegrated. About the same result will follow the action of many of the vegetable acids. It is now acknowledged by all who have given any attention to this subject, that acids are the most destructive agents that come in contact with the teeth; and that a large proportion of the caries affecting these organs, is referable directly to acids as the cause.

ALKALIES.—There are some who attribute dental degeneration to the large amount of alkalies that are used in food, at the present day, as saleratus, soda, etc. That deleterious effects upon the health of the individual may arise from the excessive use of these substances, no one will deny, but that they are peculiarly destructive to the teeth, is by no means probable, as may be proven by observation and experiment. The natural condition of the mouth, or of the fluids by which the teeth are surrounded in health, is alkaline, and it can not be supposed that a healthy secretion, normally formed and given off for the use of the animal economy, would have an injurious effect upon those structures with which it is continually in contact. If the tissues of the tooth be macerated for a long time in a strong solution of potash, no very marked effect will follow, certainly none like those which characterize the action of acids. May it not be supposed then, that these substances are not as injurious as many have been induced to believe; and if they have a degenerating effect, it is of a secondary nature, rather than primary; that they affect the dental tissues in common with other structures of the body, by interfering with the general health of the subject, and impairing the vital processes of secretion, assimilation and nutrition. It is true that statistics are presented to show that decayed and decaying teeth have been alarmingly on the increase since the introduction of saleratus as a common article of diet, but do they prove that the use of this substance is the cause of the malady? Is it not mere assumption? Could not statistics be obtained to show that intermittent fever has increased in this country, in as great a proportion since that time, as caries in the dental organs; and yet, who would argue from this alone, that they stood in the relation to each other as cause and effect? Even if the alkalies are injurious to these organs, it is never the case that they are applied in suffcient strength, or for a sufficient length of time, to produce any result whatever, and it is highly probable that under ordinary circumstances, no deleterious consequences follow their use. This is indicated by the chemical composition of the teeth, as well as by observation and experiment.

The Influence of Climate and Mode of Living.—As regards the influence of climate in hastening decay, but little is known, and that is mere hypothesis. It has been argued that persons coming from the old country generally have good teeth, but very soon after their arrival in this country, the teeth begin to decay. Is this not really owing to the manner of living rather than the climate? The aborigines of this country universally have good teeth. They live more in accordance with the laws of health than do the amalgamated mass that forms the

American people. We are continually striving to ape the vices, fashions and follies of the old world, without paying heed to their virtues, and as a consequence we enjoy the reward. The constitution is broken, the vital forces impaired, and in common with the other portions of the body, the dental organs are undergoing a process of degeneration, which will, ere long, leave its mark as well upon the intellectual faculties of the sufferer, as upon his physical organization.

SUGAR.—It has very frequently been asserted that the free use of sugar, tended to premature decay of the teeth, but at present this is rendered untenable, by the known action and influence of this substance; it being rather in an opposite direction tending to prevent instead of promoting degeneration of osseous structure. This is acknowledged by all physiologists and arguments are unnecessary. It is the principal ingredient in the food of the individual for a considerable period of his early existence, at a time, too, when these dental organs are being formed.

UNCLEANLINESS.—This is a matter to which it is the duty of every dentist and physician to call the special attention of his patients and friends. It is, indeed, the cause of all causes of premature decay. It is by uncleanliness that deleterious and obnoxious substances are allowed to remain in contact with the dental tissues, contaminating not only the teeth, but the health and life of the subject, by the foul odors that are being constantly emitted as the result of their decomposition and decay.

Persons may occasionally be found, who, for a number of years have paid no regard to cleanliness of the organs, and yet, never suffered from decaying teeth; but these are the exceptions, and not the rule, they can not be regarded as arguments in favor of uncleanliness.

In the bar-room of a small public house, in a country town, was once congregated a company of young men, enjoying the passing hours over a glass of the best "Bourbon," when an old man well known to them all as "Old Porter," joined them. "Old Porter" had for many years been the sexton of the village graveyard, and withal was an inveterate drinker. As he came in, one of the company proposed to drink the health of the old man, remarking that "Bourbon could do no one any harm, for Old Porter had drank it all his life and was then a hale and hearty old man." The old man turned abruptly to him, and requested that he walk to the churchyard. When there, the old man said, "Harry, your jesting remark brought back to my memory many a bitter thought; look there, and there," pointing to a number of graves, "I started in life with a number of young men whose prospects were as bright as yours—we drank together, enjoyed our gatherings over the social glass, as merrily as you do now; but they all are dead, and each fills a drunkard's grave. I, alone, am left. My case is not the rule, but the exception." But to the subject—we can not suppose that because one individual has reached an adult age, with perfect teeth, although he has not given any attention to the cleanliness of those organs, that others may do the same. But I have already made this article too long. What are the remedies for this degeneration? I have but one or two to present: the first, and most important is cleanliness, entire and absolute cleanliness of the whole mouth, and especially thorough washing after each meal. By this means any particles of vegetable or animal matter that may have lodged upon or around the teeth, will be removed, and their injurious effects prevented.

It will be seen by reference to the adjoining cut, that a space of greater or less dimensions exist between the teeth, into which the noxious material may easily lodge. The indentations upon the crown are not so readily seen in this representation, but any one can obtain a view of them by examining the living specimen which he carries with him. The accompaning *original* engraving is taken from a beautiful specimen, belonging to Mr. Toland, of this city; it represents, very accurately, the nervous filaments and vessels passing to the teeth, both of the superior and inferior maxilla. Reader, stop and examine it carefully. You will observe that each tooth has *five* sides, three of which may be reached with the brush—the other two, (the approximal surfaces,) must be cleansed by means of a slip of silk drawn between the teeth.

If there be a cavity, however small, have it filled; as long as it remains, it provides a resting place for a quantity of decomposing matter, which increases the decay, and taints the breath with foul and disagreeable odors.

## Figures and Tables

**Figure f1:**